# Supplementary material for: Inhibition of SGLT2 Preserves Function and Promotes Proliferation of Human Islets Cells In Vivo in Diabetic Mice
Source: Biomedicines. 2022 Jan 18;10(2):203. doi: 10.3390/biomedicines10020203 (PMC8868601; doi:10.3390/biomedicines10020203)
Supplement: Supplementary file 1 [file biomedicines-10-00203-s001.zip › biomedicines-1502298-supplementary.pdf]

biomedicines

MDPI

Supplementary Materials

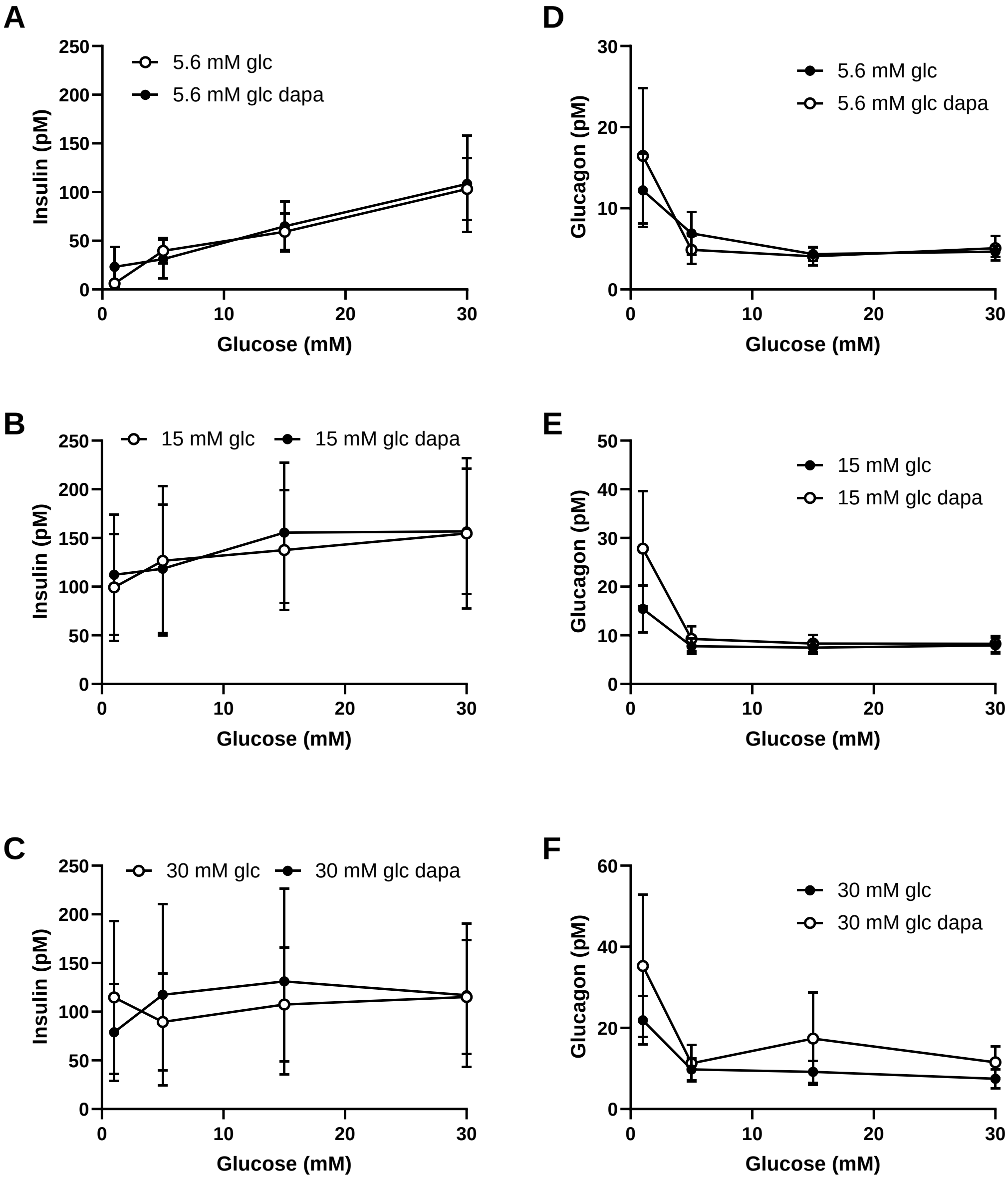

Figure S1. In vitro human islet insulin and glucagon secretion**.** Insulin and glucagon secretion from human islets cultured at 5.6 mM (**A** and **B**), 15 mM (**C** and **D**) or 30 mM (**E** and **F**) glucose with and without addition of 100 nM of dapagliflozin for 96 h. Following the 96 h incubation, islets were transferred into tubes and challenged with 1 mM, 5.6 mM, 15 mM or 30 mM glucose. Insulin and glucagon were measured after 1h incubation at 37 °C. The data was generated in three experiments with human islets from three different donors.

Table S1. Demographic data for the islet donors.

Donor ID	Age (years)	Sex (M/F)	Body Weight (kg)	BMI (kg/m²)	HbA_{1c} (%)	Experiment
HP-16148-01	43	F	52	22.4	4.9	TX
HP-16155-01	22	M	112	32.6	5.9	TX
HP-16161-01	47	M	147	38.4	5.1	TX
HP-19342-01	64	M	74	20.9	5.5	In vitro
HP-20021-01	52	M	99	29.6	5.4	In vitro
HP-20041-01	30	M	72	22.7	5.2	In vitro

The islets were isolated by Prodo Labs. Inc, Aliso Viejo, CA, USA. A unique identifier number is given for each donor. There was no reported history of diabetes in any of the subjects. Islets from three donors were used for transplantation (TX) and islets from three other donors were used for the in vitro experiments.

Table S2. Immunohistological evaluation of pancreas from non-diabetic control mice, diabetic control mice and diabetic mice treated with dapagliflozin.

	**Non-Diabetic (*n* = 6)**	**Diabetic Vehicle (*n* = 21)**	**Diabetic Dapagli-flozin (*n* = 17)**
$Insulin^+$ (mm^2)	0.048 ± 0.031	0.015 ± 0.009 **	0.024 ± 0.025 *
$Glucagon^+$ (mm^2)	0.0015 ± 0.0007	0.021 ± 0.010 **	0.027 ± 0.026 **
$Insulin^+$/islet (%)	97 ± 1.6	40±14 ***	45 ± 26 ***
$Glucagon^+$/islet (%)	3.4 ± 1.6	60 ± 14 ***	55 ± 26 ***
Pancreatic islet area (Ins+ and Gcg+)/pancreas (%)	0.13 ± 0.08	0.11 ± 0.052	0.16 ± 0.12

The results are presented as mean ± SD and statistical significance was determined using Kruskal-Wallis test, followed by Dunn's multiple comparison test for differences between the groups. * $P < 0.05$, ** $P < 0.01$, *** $P < 0.001$ for significant difference compared to the non-diabetic group. Pancreatic islet area was calculated from immunohistochemical staining as the $insulin^+$ and $glucagon^+$ area divided by the total pancreas area.

Table S3. Comparison of mice with high versus low alpha and beta cell proliferation.

	**High Proliferation *n* = 8**	**Low Proliferation *n* = 10**	***P*-value**
Glucose at termination (mM)	15.0 ± 2.5	18.7 ± 2.4	ns
$AUC_{glc\ (Day\ 0–53)}$ (mM × days)	729 ± 76	762 ± 73	ns
IVArgGTT parameters			
$AUC_{glc\ (1–10\ min)}$ (mM × min)	158 ± 19	138 ± 12	ns
$AUC_{ins\ (1–10\ min)}$ (ng/ml × min)	3.26 ± 0.83	3.89 ± 0.79	ns
Human C-peptide (pM)	238 ± 83	373 ± 114	ns
Fructoseamine (μM)	216 ± 11	247 ± 11	ns
Glucagon (pM)	36.2 ± 4.8	42.0 ± 3.6	ns
Histological analysis of the grafted islets			
% insulin$^+$ cells	49.3 ± 1.6	60.9 ± 2.4	0.0004
% glucagon$^+$ cells	43.7 ± 2.0	37.2 ± 2.3	0.04
% Ki67$^+$insulin$^+$ cells	1.5 ± 0.2	0.48 ± 0.08	0.004
% Ki67$^+$glucagon$^+$ cells	5.5 ± 1.3	1.4 ± 0.2	0.01
% PDX-1$^+$glucagon$^+$	0.22 ± 0.09	0.13 ± 0.03	ns

The high proliferation group was set to ≥2.5% Ki67$^+$glucagon$^+$ cells and/or ≥1% Ki67$^+$insulin$^+$ cells. The low proliferation group represented mice with <2.5% Ki67$^+$glucagon$^+$ cells and <1% Ki67$^+$insulin$^+$ cells. Data are shown as mean ± SEM. Statistical difference between the two groups were determined with Student's t-test. ns = not significant
